# Single-Session Pulsed-Field Ablation Combined With Transcatheter Edge-to-Edge Repair for Atrial Fibrillation and Mitral Regurgitation: A Prospective Ten-Patient Series

**DOI:** 10.1016/j.shj.2026.100836

**Published:** 2026-03-19

**Authors:** Alica Cesnakova Konecna, Otakar Jiravsky, Jan Chovancik, Miroslav Hudec, Jaroslav Januska, Libor Gajdusek, Tomas Kubat, Ivan Ranic, Bogna Jiravska Godula, Jan Alexander Mohr, Leos Pleva

**Affiliations:** a AGEL Hospital Trinec-Podlesi, Trinec, Czech Republic; b Faculty of Medicine, University of Ostrava, Ostrava, Czech Republic; c Faculty of Medicine, Masaryk University, Brno, Czech Republic

**Keywords:** Atrial fibrillation, Combined procedure, Mitral regurgitation, Pulsed-field ablation, Transcatheter edge-to-edge repair

## Abstract

• This research correspondence examines the first expanded cohort combining pulsed-field ablation (PFA) with transcatheter edge-to-edge repair via single transseptal access.
• Sinus rhythm was 90% at 6 months with significant New York Heart Association improvement (p = 0.008).
• No strokes, tamponade, or PFA-related complications were experienced in the high-risk cohort.
• PFA tissue selectivity enables safe combination with mitral transcatheter edge-to-edge repair.
• The median procedure time of 102 minutes supports the feasibility of a one-stop approach.

## Graphical abstract

**Figure:**
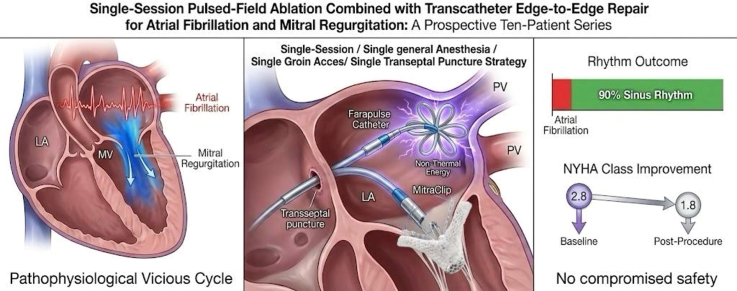

Atrial fibrillation (AF) affects approximately 50% of patients undergoing transcatheter edge-to-edge repair (TEER) and is associated with substantially worse outcomes, including 55% higher mortality and 30% higher heart-failure hospitalization rates.^1^ These conditions share bidirectional pathophysiology (Graphical Abstract): AF promotes left atrial (LA) and annular dilatation that worsens mitral regurgitation (MR), whereas MR creates atrial stretch that perpetuates AF. Addressing MR alone may therefore not fully optimize outcomes. Pulsed-field ablation (PFA) has emerged as an attractive AF treatment due to its tissue selectivity, with no esophageal complications, pulmonary vein stenosis, or persistent phrenic nerve palsy among >17,000 patients in the MANIFEST-17K study.^2^ The surgical Cardiothoracic Surgical Trials Network trial established that concomitant AF ablation during mitral valve surgery significantly improves sinus rhythm maintenance (63.2 vs. 29.4% at 12 months, *p* < 0.001) without increasing complications.^3^ Building on the first-in-human combined PFA-TEER case,^4^ we report a prospective 10-patient series evaluating single-session PFA with MitraClip (Abbott, Santa Clara, CA) TEER.

We enrolled 10 consecutive high-surgical-risk patients with symptomatic AF and moderate-to-severe MR at a single center between December 2023 and March 2025. Eligibility required paroxysmal or persistent AF with suitable anatomy for catheter ablation and severe symptomatic MR meeting contemporary TEER criteria. MR etiology was secondary (functional) in eight patients (80%) and mixed in two (20%). Ethics approval was obtained (EK-23-046).

All procedures used a single transseptal puncture via a modified Brockenbrough technique under transesophageal echocardiography guidance. The puncture site was targeted at the midfossa ovalis, approximately 4.0 to 4.5 cm above the mitral annulus plane—a "universal" height accommodating both the Faradrive steerable sheath (Boston Scientific, Marlborough, MA) for PFA delivery and subsequently the MitraClip guide catheter through wire exchange. PFA achieved complete pulmonary vein isolation in all patients. Additional substrate modification including posterior wall ablation was performed in eight patients (80%), with ablation strategy individualized based on AF type and substrate characteristics. Postablation direct-current cardioversion (DCCV) was performed in patients remaining in AF.

Baseline characteristics are shown in Table 1 (individual patient data in Supplementary Table 1). The median age was 76 years (range 65-84), 60% were women, and AF was persistent in 60%. The mean CHA_2_DS_2_-VASc score was 4.0 ± 1.4, reflecting substantial thromboembolic risk. Patients were functionally limited with mean New York Heart Association class of 2.8 ± 0.4, mean left ventricular ejection fraction of 44% ± 15%, and mean LA diameter of 48.5 mm ± 5.7 mm. Six patients (60%) were receiving amiodarone at baseline. Two patients (20%) underwent concomitant TriClip for significant tricuspid regurgitation, representing a more advanced heart failure phenotype.

**Table 1:** Baseline characteristics, procedural data, and outcomes (N = 10)

Baseline characteristics	
Age, y, median (range)	76 (65-84)
Female sex, n (%)	6 (60)
AF type: paroxysmal/persistent, n	4/6
MR etiology: secondary/mixed, n	8/2
NYHA class, mean ± SD	2.8 ± 0.4
LVEF, %, mean ± SD	44 ± 15
Baseline amiodarone, n (%)	6 (60)
Concomitant TriClip, n (%)	2 (20)
Procedural data	
Procedure time, min, median (IQR)	102 (95-130)
Ablation strategy, n (%)	
PVI only	2 (20)
PVI + posterior wall isolation	2 (20)
Complex (PVI + PWI + linear ablations)	6 (60)
Postablation DCCV, n (%)	9/10 (90)
Technical success (MR ≤ 2+), n (%)	7/10 (70)
Technical success excl. TriClip, n (%)	7/8 (87.5)
Rhythm outcomes	
Discharge: sinus rhythm, n/N (%)	8/10 (80)
M1: sinus rhythm, n/N (%)	7/9 (78)
M3: sinus rhythm, n/N (%)∗	6/9 (67)
M6: sinus rhythm, n/N (%) [95% CI]	9/10 (90) [56-100]
M12: sinus rhythm, n/N (%)	5/5 (100)
Repeat DCCV during follow-up, n (%)	1/10 (10)
Extended monitoring (Holter/ICD), n (%)	7/10 (70)
AF burden, %, mean (median, range)	6 (0, 0-32)
Six-month clinical outcomes	
NYHA class, mean ± SD	1.8 ± 0.6 (*p* = 0.008)†
MR ≤2+/2-3+/≥3, n (%)	3 (33)/2 (22)/4 (44)
NT-proBNP, pg/mL, mean ± SD	1664 ± 1334
Safety events	
Device complication (partial clip dislocation)	1 (10)
Retroperitoneal bleed (related to unknown tumor)	1 (10)

Continuous variables are summarized as mean ± SD or median (range/IQR); paired within-patient comparisons between baseline and follow-up were performed using the paired Student's t-test, with Cohen's d reported as the standardized effect size.

Abbreviations: AF, atrial fibrillation; DCCV, direct-current cardioversion; ICD, implantable cardioverter-defibrillator; IQR, interquartile range; LVEF, left ventricular ejection fraction; M1/3/6/12, month 1/3/6/12; MR, mitral regurgitation; NT-proBNP, N-terminal pro-B type natriuretic peptide; NYHA, New York Heart Association; PVI, pulmonary vein isolation; PWI, posterior wall isolation.

∗ One patient required repeat DCCV at M3.

† p-value reflects the within-patient change from baseline to 6-month follow-up (n = 9 paired observations), assessed by paired Student's t-test on numerically coded NYHA class. Cohen's d = 1.15.

All 10 procedures were successfully completed via single transseptal access with a median procedure time of 102 minutes (interquartile range: 95-130). Postablation DCCV was required in 9/10 patients (90%); 1 patient with persistent AF presented in sinus rhythm at baseline. Acute technical success (MR reduced to ≤2+) was achieved in 7/10 patients (70%), below contemporary TEER registry benchmarks of 85% to 95%.^5^ However, both TriClip recipients had suboptimal acute results; with exclusion of these patients with concomitant tricuspid intervention, technical success was 87.5% (7/8), consistent with published benchmarks.

Clinical follow-up with 12-lead electrocardiogram (ECG) was performed at discharge and 1, 3, 6, and 12 months postprocedure (M1/3/6/12). At discharge, 8/10 (80%) were in sinus rhythm. Rhythm control showed characteristic blanking-period nadir at M3 (6/9, 67%) before improving (Supplementary Figure 1). One patient (P4) required repeat DCCV at M3 for AF recurrence and subsequently maintained sinus rhythm. By M6, 9/10 (90%; 95% CI: 56%-100%) were in sinus rhythm. At M12, all 5 patients with available data (100%) (including P6, who converted from AF at M6 without reablation but required propafenone) maintained sinus rhythm (Supplementary Table 2).

Extended rhythm monitoring with Holter ECG or implantable cardioverter-defibrillator interrogation was available in seven patients (70%). The mean AF burden was 6% (median 0%, range 0%-32%). Five patients demonstrated 0% AF burden; 1 (P8) had 10% AF burden on Holter and 1 with an cardioverter-defibrillator (P2) showed 32% AF burden despite sinus rhythm on 12-lead ECG, illustrating the limitation of snapshot monitoring. Three patients lacked quantitative AF burden data.

New York Heart Association class improved significantly from 2.8 ± 0.4 to 1.8 ± 0.6 (*p* = 0.008; Cohen's d = 1.15), representing clinically meaningful functional improvement consistent with major TEER trials. Postclip, mean LA pressure decreased from 13.5 ± 6.3 to 11.6 ± 5.4 mmHg (*p* = 0.36), a modest nonsignificant reduction reflecting competing effects of MR reduction and clip-induced transmitral gradient changes. For nine patients, MR grade was as follows at M6: ≤2+ in 3 (33%), 2 to 3+ in 2 (22%), and ≥3+ in 4 (44%) (Supplementary Table 3). LA diameter was unchanged (48.5 vs. 49.7 mm), consistent with the 12 to 24 month timeline typically required for reverse remodeling. N-terminal pro-B type natriuretic peptide decreased from 2573 ± 1914 to 1664 ± 1257 pg/mL (Supplementary Table 4).

One device complication occurred: partial clip dislocation at 6-month echocardiography (P7, TriClip recipient), managed conservatively with acceptable residual regurgitation. One retroperitoneal hemorrhage from an incidentally discovered renal tumor was treated with selective arterial embolization; although anatomically unrelated to procedural access, the temporal relationship is acknowledged. Consistent with the favorable safety profile of PFA's tissue selectivity, no strokes, cardiac tamponade, esophageal injury, or phrenic nerve palsy occurred.

Several limitations warrant acknowledgment. This small exploratory series is underpowered for formal efficacy conclusions. Rhythm monitoring relied primarily on snapshot 12-lead ECGs, likely underestimating asymptomatic AF recurrence; extended monitoring was available in only 70% of patients. Continued antiarrhythmic therapy in 60% of patients confounds assessment of ablation-specific effects. The two TriClip patients introduced procedural heterogeneity, although sensitivity analysis excluding them showed 87.5% technical success; future studies should either exclude concomitant tricuspid intervention or analyze such patients separately. The absence of LA reverse remodeling at 6 months may reflect insufficient follow-up duration rather than treatment failure.

With 90% sinus rhythm on ECG at 6 months, mean 6% AF burden on extended monitoring, and significant functional improvement, single-session PFA combined with TEER through single transseptal puncture was technically feasible and safe in this high-surgical-risk cohort. The tissue selectivity of PFA makes it well suited for combination with TEER. These preliminary findings support evaluation of this "one-stop" approach in larger multicenter studies with systematic continuous rhythm monitoring and formal heart-failure endpoint assessment.

## CRediT Authorship Contributions

Alica Cesnakova Konecna and Otakar Jiravsky conceived and designed the study, curated the data, and wrote the original draft of the manuscript. Jan Chovancik, Miroslav Hudec, Jaroslav Januska, Libor Gajdusek, and Ivan Ranicperformed the procedures and collected the clinical data. Tomas Kubat contributed to data curation and performed the statistical analysis. Bogna Jiravska Godula and Jan Alexander Mohr analyzed the data and provided critical revision of the manuscript. Leos Pleva supervised the project and contributed to writing, review, and editing. All authors have read and approved the final version of the manuscript.

## Ethics Statement

This study was conducted in accordance with the Declaration of Helsinki. The protocol was approved by the Ethics Committee of AGEL Hospital Trinec-Podlesi (EK-23-046).

## Consent Statement

All patients provided written informed consent for the procedure and registry participation.

## Funding

The authors have no funding to report.

## Data Availability

Data available from the corresponding author on reasonable request.

## Disclosure Statement

The authors report no conflict of interest.

## Supplementary Material

Supplemetary Figures
