# Supplementary figures and images for: Single-Session Pulsed-Field Ablation Combined With Transcatheter Edge-to-Edge Repair for Atrial Fibrillation and Mitral Regurgitation: A Prospective Ten-Patient Series
Source: Struct Heart. 2026 Mar 19;10(7):100836. doi: 10.1016/j.shj.2026.100836 (PMC13224063; doi:10.1016/j.shj.2026.100836)

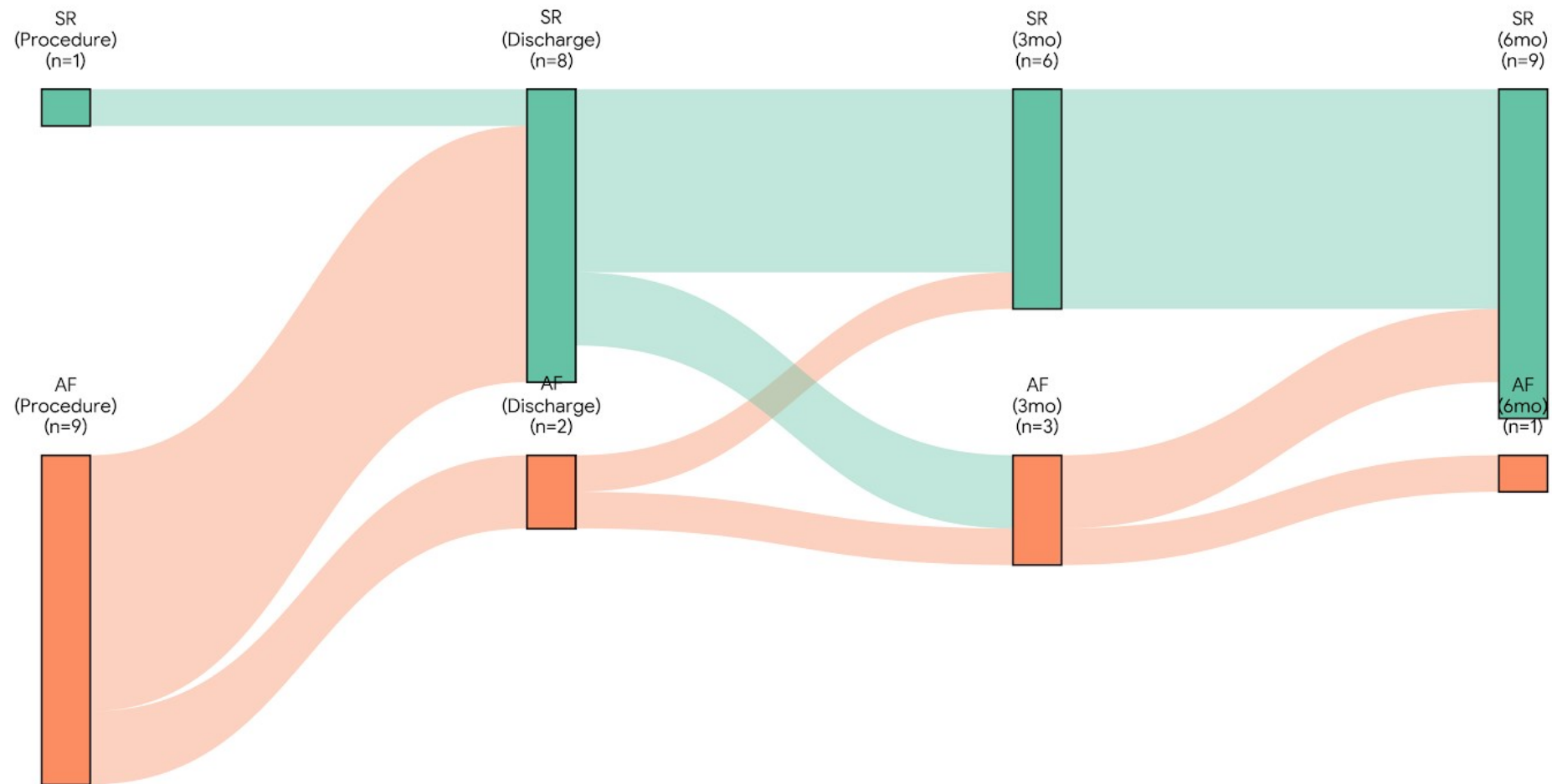

Supplement: Supplemetary Figures [file mmc1.pdf]
