# Supplementary material for: Single-Session Pulsed-Field Ablation Combined With Transcatheter Edge-to-Edge Repair for Atrial Fibrillation and Mitral Regurgitation: A Prospective Ten-Patient Series
Source: Struct Heart. 2026 Mar 19;10(7):100836. doi: 10.1016/j.shj.2026.100836 (PMC13224063; doi:10.1016/j.shj.2026.100836)
Supplement: Supplementary Tables 1-4 [file mmc2.docx]

**SUPPLEMENTARY MATERIALS**

*Single-Session Pulsed-Field Ablation Combined with Transcatheter Edge-to-Edge Repair for Atrial Fibrillation and Mitral Regurgitation*

**Table S1. Individual Patient Baseline Characteristics and Procedural Details**

| **Pt** | **Age** | **Sex** | **BMI** | **AF Type** | **CHA₂DS₂-VASc** | **LVEF (%)** | **LA (mm)** | **BL MR** | **Ablation Strategy** | **Proc Time (min)** | **Fluoro (min)** | **PFA Apps** | **Clips** | **DCCV** |
| --- | --- | --- | --- | --- | --- | --- | --- | --- | --- | --- | --- | --- | --- | --- |
| P1 | 77 | M | 21.7 | Persistent | 4 | 45–50 | 49 | 4/4 | Complex | 130 | 50 | 89 | 1 | Yes |
| P2 | 76 | M | 27.1 | Persistent | 4 | 15–20 | 57 | 4/4 | Complex | 145 | 60 | 83 | 2 | Yes |
| P3 | 67 | F | 27.6 | Paroxysmal | 7 | 50 | 44 | 4/4 | Complex | 190 | 55 | 52 | 1 | Yes |
| P4 | 84 | F | 23.8 | Persistent | 3 | 60 | 51 | 4/4 | Complex | 100 | 75 | 78 | 1 | Yes |
| **P5†** | 65 | M | 33.7 | Persistent | 3 | 30–35 | 54 | 4/4 | PVI+PWI | 95 | 60 | 59 | 1 | No |
| **P6‡** | 75 | F | 34.2 | Persistent | 4 | 55 | 41 | 2–3/4 | Complex | 95 | 19 | 81 | 1 | Yes |
| P7 | 73 | F | 32.5 | Persistent | 2 | 55 | 55 | 3–4/4 | Complex | 130 | 39 | 81 | 1+T | Yes |
| P8 | 76 | F | 23.0 | Paroxysmal | 3 | 60 | 44 | 4/4 | PVI+PWI | 105 | 25 | 52 | 1+T | Yes |
| P9 | 74 | F | 28.0 | Paroxysmal | 4 | 45 | 40 | 4/4 | PVI | 85 | 31 | 50 | 1 | Yes |
| P10 | 76 | M | 29.1 | Paroxysmal | 6 | 20 | 50 | 4/4 | PVI | 75 | 33 | 52 | 1 | Yes |

*AF = atrial fibrillation; BL = baseline; DC = discharge; M1/3/6/12 = months post-procedure; NYHA = New York Heart Association; PVI = pulmonary vein isolation; PWI = posterior wall isolation; SR = sinus rhythm. *Cardioverted at 3 months. †Best outcome: SR at baseline, no DCCV, greatest improvement. ‡Only patient in AF at 6 months. — = data not available.*

**Table S2. Individual Patient Rhythm Trajectory and Clinical Outcomes**

| **Pt** | **AF Type** | **Baseline** | **Ablation** | **DC** | **M1** | **M3** | **M6** | **M12** | **BL NYHA** | **6mo NYHA** | **ΔNYHA** |
| --- | --- | --- | --- | --- | --- | --- | --- | --- | --- | --- | --- |
| P1 | Persistent | AF | Complex | AF | SR | SR | SR | SR | III | I | −2.0 |
| P2 | Persistent | AF | Complex | SR | SR | SR | SR | SR | II-III | I-II | −1.0 |
| P3 | Paroxysmal | AF | Complex | SR | SR | --- | SR | SR | III | II | −1.0 |
| P4 | Persistent | AF | Complex | SR | SR | AF* | SR | --- | III | --- | --- |
| **P5†** | Persistent | SR | PVI+PWI | SR | SR | SR | SR | SR | III-IV | I | −2.5 |
| **P6‡** | Persistent | AF | Complex | SR | AF | AF | AF | SR | III | III | 0.0 |
| P7 | Persistent | AF | Complex | AF | AF | AF | SR | --- | II | II | 0.0 |
| P8 | Paroxysmal | AF | PVI+PWI | SR | --- | SR | SR | --- | II-III | II | −0.5 |
| P9 | Paroxysmal | AF | PVI | SR | SR | SR | SR | --- | II-III | II | −0.5 |
| P10 | Paroxysmal | AF | PVI | SR | SR | SR | SR | --- | III | I-II | −1.5 |

*AF = atrial fibrillation; BL = baseline; DC = discharge; M1/3/6/12 = months post-procedure; NYHA = New York Heart Association; PVI = pulmonary vein isolation; PWI = posterior wall isolation; SR = sinus rhythm. *Cardioverted at 3 months. †Best outcome: SR at baseline, no DCCV, greatest improvement. ‡Only patient in AF at 6 months.*

**Table S3. Individual Patient Hemodynamic and Echocardiographic Data**

| **Pt** | **LA-P Pre*** | **LA-P Post*** | **ΔLA-P** | **BL LA (mm)** | **6mo LA (mm)** | **BL LVEF (%)** | **6mo LVEF (%)** | **BL LVEDD (mm)** | **6mo LVEDD (mm)** | **DC MR** | **6mo MR** |
| --- | --- | --- | --- | --- | --- | --- | --- | --- | --- | --- | --- |
| P1 | 10 | 7 | −3 | 49 | 48 | 45–50 | 55 | 52 | 55 | 2 | 2–3 |
| P2 | 26 | 23 | −3 | 57 | 59 | 15–20 | 15–20 | 70 | 70 | 1–2 | 2–3 |
| P3 | 19 | 14 | −5 | 44 | 49 | 50 | 45–50 | 53 | 56 | 3 | 3–4 |
| P4 | 14 | 8 | −6 | 51 | --- | 60 | --- | 53 | --- | 1–2 | --- |
| **P5†** | 6 | 7 | +1 | 54 | 49 | 30–35 | 30 | 67 | 67 | 2 | 1 |
| **P6‡** | 18 | 19 | +1 | 41 | 44 | 55 | 55 | 42 | 46 | 1–2 | 3–4 |
| P7 | 16 | 9 | −7 | 55 | 51 | 55 | 55 | 55 | 54 | 2–3 | 4 |
| P8 | 9 | 10 | +1 | 44 | 49 | 60 | 60–70 | 52 | 43 | 2–3 | 3 |
| P9 | 4 | 6 | +2 | 40 | 42 | 45 | 45–50 | 58 | 50 | 1 | 2 |
| P10 | 13 | 13 | 0 | 50 | 55 | 20 | 20–25 | 62 | 63 | 2 | 2 |

*BL = baseline; DC = discharge; LA = left atrial; LA-P = left atrial pressure (median of 3 measurements, mmHg); LVEDD = left ventricular end-diastolic diameter; LVEF = left ventricular ejection fraction; MR = mitral regurgitation; 6mo = 6 months. *LA pressure values represent median of three measurements before and after clip deployment. †Best responder with greatest hemodynamic benefit. ‡Only patient in AF at 6 months. --- = data not available.*

**Table S4. NT-proBNP Changes and Rhythm Status**

| **Patient** | **Baseline NT-proBNP (pg/mL)** | **6-mo NT-proBNP (pg/mL)** | **ΔNT-proBNP (pg/mL)** | **6-mo Rhythm** | **ΔNYHA** |
| --- | --- | --- | --- | --- | --- |
| P1 | 1668 | 596 | −1072 | SR | −2.0 |
| P2 | 2226 | 2093 | −133 | SR | −1.0 |
| P3 | 7055 | 2468 | −4587 | SR | −1.0 |
| P4 | 4291 | --- | --- | SR | --- |
| **P5†** | 1630 | 309 | −1321 | SR | −2.5 |
| **P6‡** | 2338 | 3038 | +700 | AF | 0.0 |
| P7 | 1318 | 579 | −739 | SR | 0.0 |
| P8 | 2631 | 795 | −1836 | SR | −0.5 |
| P9 | 859 | 959 | +100 | SR | −0.5 |
| P10 | 1712 | 4135 | +2423 | SR | −1.5 |
| Mean±SD | 2573±1914 | 1664±1257 | −718±2053 | --- | −1.0±0.9 |

*BL = baseline; DC = discharge; LA = left atrial; LA-P = left atrial pressure (median of 3 measurements, mmHg); LVEDD = left ventricular end-diastolic diameter; LVEF = left ventricular ejection fraction; MR = mitral regurgitation; 6mo = 6 months. *LA pressure values represent median of three measurements before and after clip deployment. †Best responder with greatest hemodynamic benefit. ‡Only patient in AF at 6 months.* --- *= data not available.*
